# New opportunities for designing effective small interfering RNAs

**DOI:** 10.1038/s41598-019-52303-5

**Published:** 2019-11-06

**Authors:** James J. Valdés, Andrew D. Miller

**Affiliations:** 1 Veterinary Research Institute, Hudcova 70, CZ-62100 Brno, Czech Republic; 2 Institute of Parasitology, Biology Centre, Czech Academy of Sciences, Branišovska 1160/31, CZ-37005 České Budějovice, Czech Republic; 3 KP Therapeutics Ltd, 86 Deansgate, Manchester, M3 2ER UK

**Keywords:** Molecular modelling, Biochemical reaction networks, Structure-based drug design

## Abstract

Small interfering RNAs (siRNAs) that silence genes of infectious diseases are potentially potent drugs. A continuing obstacle for siRNA-based drugs is how to improve their efficacy for adequate dosage. To overcome this obstacle, the interactions of antiviral siRNAs, tested *in vivo*, were computationally examined within the RNA-induced silencing complex (RISC). Thermodynamics data show that a persistent RISC cofactor is significantly more exothermic for effective antiviral siRNAs than their ineffective counterparts. Detailed inspection of viral RNA secondary structures reveals that effective antiviral siRNAs target hairpin or pseudoknot loops. These structures are critical for initial RISC interactions since they partially lack intramolecular complementary base pairing. Importing two temporary RISC cofactors from magnesium-rich hairpins and/or pseudoknots then kickstarts full RNA hybridization and hydrolysis. Current siRNA design guidelines are based on RNA primary sequence data. Herein, the thermodynamics of RISC cofactors and targeting magnesium-rich RNA secondary structures provide additional guidelines for improving siRNA design.

## Introduction

The discovery of RNA interference (RNAi)^1^ is seminal to understanding post-transcriptional modulation due to its selectivity and specificity in silencing the target messenger RNA (mRNA). Over the past decade, preclinical and clinical investigations suggest RNAi effectors as therapy for human diseases. Once *in vivo* dosage and delivery problems are solved these RNAi effectors will revolutionize precise therapeutic approaches^2–5^. A genuine RNAi effector against human diseases is small interfering RNA (siRNA) - a class of A-form double-stranded RNAs that are 19–23 bp long. The eight guidelines specified for designing effective siRNAs are based on the primary nucleotide (nt) sequence (see Reynolds *et al*.^6^ for details). Since many experimental studies test the efficacy of synthesized antiviral siRNAs^7–14^, extensive *ex post facto* investigations will therefore provide additional design guidelines for improving siRNA dosages.

**Figure 1:**
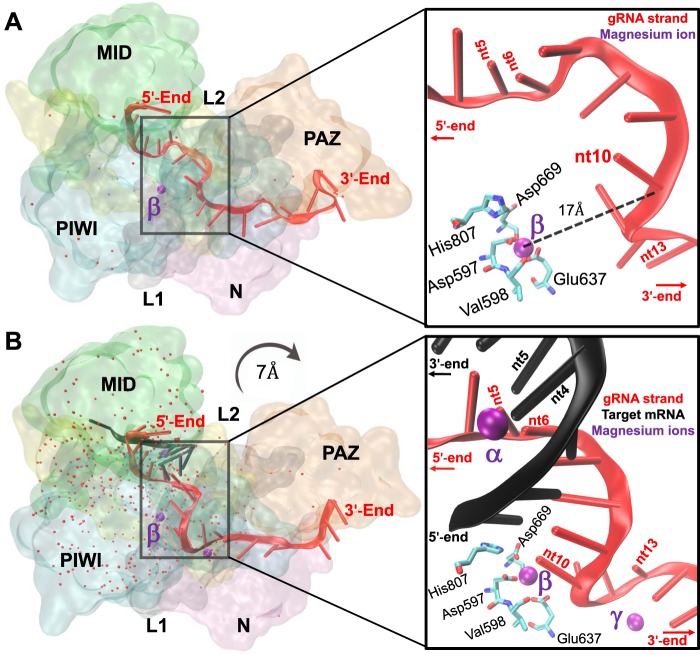
The RISC structures. The optimized pre-RISC (**A**) (PDB: 4W5N) and post-RISC structures (**B**) (PDB: 4W5O) are shown with Ago2 in surface representation and its domains color-labeled. The Ago2 domains are the amino-terminus (N), P element-induced wimpy testis (PIWI), middle (MID), Piwi–Argonaute–Zwille (PAZ), and the linkers (L) that connect the MID-PIWI and N-PAZ lobes. The structures include the gRNA (red ribbon), the target mRNA (black ribbon; only in the post-RISC) (**B**), the water molecules (red oxygen points) and the magnesium cofactors (purple spheres). The labeled magnesium-β and gRNA 5′- and 3′-end are color-labeled respectively. The zoomed insets (right; oriented for better viewing) show the labeled magnesium cofactors (purple spheres) and proximate nucleotide residues (nt) that are color-labeled accordingly - gRNA (red) and mRNA (black; lower inset). The labeled PIWI residues that coordinate magnesium-β are indicated.

The RNAi process begins when synthetic, or *in situ* generated, siRNAs are transformed in all cells as active participants of the RNA-induced silencing complex (RISC). Central to the RISC catalytic cycle is siRNA binding to the nuclease argonaute 2 (Ago2). From the amino-terminus to the carboxyl-terminus, the Ago2 domains are designated N, L1, PAZ, L2, MID and PIWI (Fig. 1). As described by Nakanishi^15^, the Ago2 MID-PIWI nt pocket initially binds the 5′-terminus of the siRNA antisense strand, or guide strand (gRNA). The α−7 helix of the L2 domain then displaces the siRNA duplex separating the sense strand, or passenger strand (pRNA). This strand separation facilitates the full gRNA to thread through Ago2. The gRNA 3′-terminus then binds to the flexible PAZ domain that ejects the pRNA altogether from the RISC. At this stage, the solvent exposed positioning of gRNA nt2-nt4 of the seed region (nt2-nt8) is critical for initial complementary mRNA base pairing^15^. From herein, such an Ago2-gRNA bound conformation is known as the pre-activated RISC (pre-RISC) (Fig. 1A).

Full gRNA-mRNA hybridization is triggered once the gRNA critical seed region (nt2-nt4) binds to the target mRNA. The fully hybridized structure is herein termed the activated-RISC^16^. The target mRNA is then hydrolyzed positionally at the 5′-phosphodiester bond whose flanking nt residues complement gRNA nt10-nt11^17^. Although X-ray crystal structures of the human activated-RISC are not resolved, Schirle *et al*.^16^ were able to generate a RISC structure after complementary mRNA hydrolysis. The post-activated RISC (post-RISC) contains an A-form double-helix made up of the gRNA seed region (nt2-nt8) hybridized to its complementary mRNA (Fig. 1B). The post-RISC mRNA 3′-terminus is not complemented by the gRNA 5′-terminus, but is coordinated by Ser561 between the MID and L2 domains^16^. The hydration difference between the pre- and post-RISC (Fig. 1) indicates that RNA-associated water molecules are imported during the activated-RISC stage. A conformational difference between the pre- and post-RISC structures is indicated by the 7 Å widening of the N-PAZ channel caused by the flexible PAZ domain (Fig. 1).

The RISC catalytic cycle involves one persistent cofactor (magnesium-β) and two temporary cofactors (magnesium-α and magnesium-γ) (Fig. 1). The difference in magnesium persistence and ion count between the pre- and post-RISC suggests three functional hypotheses (i-iii). The first hypothesis indicates that (i) the persistent presence of magnesium-β determines siRNA efficacies by the strength of Ago2-gRNA-mRNA interactions. The second and third hypotheses are linked, demonstrating that (ii) effective gRNA-mRNA interactions are initiated and consolidated by magnesium-rich mRNA secondary structures^18^. These mRNA secondary structures therefore enable (iii) the RISC catalytic cycle as the primary source for importing magnesium-α and magnesium-γ. Accordingly, several computational and molecular analyses were performed to investigate these three hypotheses and their potential contributions to siRNA design.

## Results

### Thermodynamics of the RISC and its cofactors

**Figure 2:**
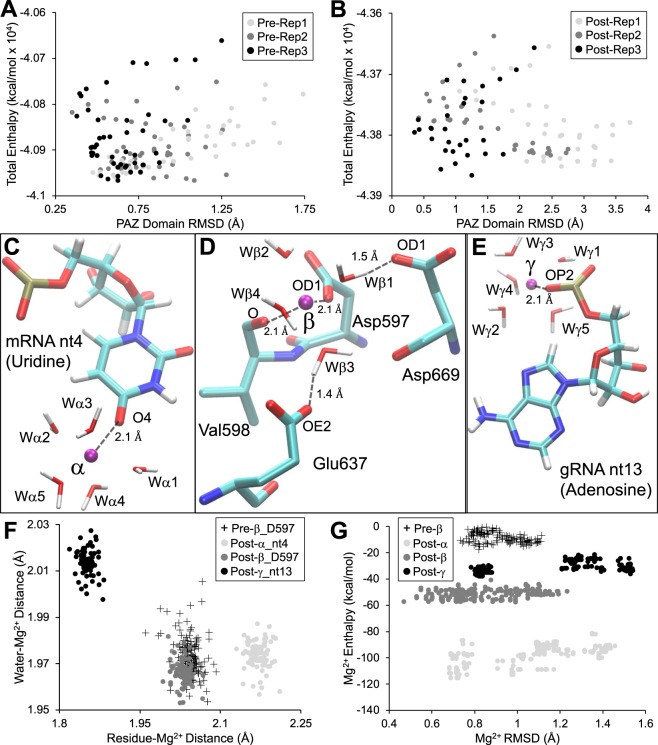
Thermodynamics of the RISC and its cofactors. The top panels are the total enthalpy (kcal/mol; y-axis) and PAZ-domain RMSD (x-axis) for each replicate (Rep) of the pre- (**A**) and post-RISC (**B**). Panels C-E show the ligating residues (atoms labeled) and inner shell water molecules (Wα, Wβ and Wγ) that coordinate each respective RISC cofactor (purple spheres and labeled). Distance contacts are shown as dashed lines. The lower panels (**F,G**) include pre-RISC measurements of magnesium-β (+). Magnesium contact distances with water molecules (y-axis) and ligating residues (x-axis) are depicted (**F**). For clarity, only contacts between magnesium-β and Asp597 are shown (**F**). The additional magnesium-β contacts are in Supplementary Figure S1. The RMSD (x-axis) and enthalpy (kcal/mol; y-axis) for each cofactor are shown for the pre- and post-RISC (**G**).

Stochastic molecular simulations were conducted to test hypothesis (i) using the pre- and post-RISC structures (Fig. 1). The difference in total enthalpy of each replicate during the simulations (Fig. 2A-B) is negligible for the pre-RISC (*η*^2^ = 0.006) and minimal for the post-RISC (*η*^2^ = 0.04) (see Supplementary Table S1 and S2 for descriptive statistics). The presence/absence of magnesium cofactors and of the target mRNA (Fig. 1), however, cause a distinction in the average total enthalpy between the pre-RISC (−40899 ± 6 kcal/mol; *η*^2^ = 1) and the post-RISC (−43804 ± 46 kcal/mol; *η*^2^ = 1). The two RISC structures also vary in the flexibility of the PAZ-domain (*η*^2^ = 0.38). The α-carbon backbone of the post-RISC PAZ-domain has a root mean square deviation (RMSD) of 1.81 ± 0.9 Å during the simulations, which is greater than the pre-RISC (RMSD: 0.8 ± 0.3 Å) (Fig. 2A,B). The remaining Ago2 domains exhibit lower deviations in both the pre-RISC (RMSD: 0.6 ± 0.1 Å) and post-RISC (RMSD: 0.8 ± 0.3 Å) (Supplementary Fig. S1A).

As a divalent cation, a tetrahydrated nt-bound magnesium has a low energy state, but energy decreases further when the inner solvation shell is occupied by six water molecules (hexahydrated)^19^. These inner shell water molecules maintain a distance of ~2 Å from the magnesium ion^19^. The post-RISC magnesium-α and magnesium-γ are pentahydrated, but the pre- and post-RISC magnesium-β are hexahydrated (Fig. 2C–E). The simulations show that the hydrating water molecules of magnesium-α and magnesium-β (pre- and post-RISC) maintain a distance of 1.97 ± 0.01 Å, while the water distances of magnesium-γ is 2.01 ± 0.01 Å (Fig. 2F). The pre- and post-RISC magnesium-β, however, show slight deviations between the two types of inner hydration molecular environments (Supplementary Table S3; *η*^2^ = 0.051). Outer shell water molecules of magnesium-β show smaller deviations between the pre- and post-RISC (Supplementary Table S3; *η*^2^ = 0.018).

Contact distances of magnesium coordinating residues were also calculated to further verify that the stochastic molecular simulations comport with crystallographic results (Fig. 2C–E). At the inner cofactor shell, magnesium-α binds to mRNA nt4, magnesium-γ binds to gRNA nt13, and magnesium-β is coordinated by the conserved Asp-Glu-Asp catalytic triad (Fig. 1 insets and Fig. 2C–E). Only Asp597 of the catalytic triad, along with Val598, form inner shell contacts with magnesium-β (Fig. 2D). Both Glu637 and Asp669 of the triad form second shell contacts with magnesium-β via intermediate inner shell water molecules (Fig. 2D).

The contact distances for the ligating residues of each cofactor during the simulations (Fig. 2F and Supplementary Fig. S1B-S1D) are in good agreement to those in the crystal structure (Fig. 2C–E). The conformation of Glu637 is flexible during the RISC cycle due to its pivotal role in mRNA hydolysis^20^. This conformational flexibility explains the deviation of Glu637 in the post-RISC (3.3 ± 0.09 Å) compared to the pre-RISC (1.8 ± 0.02 Å) (Supplementary Fig. S1C). The decreased distance between magnesium-γ and gRNA nt13 (Fig. 2F) was also previously reported in molecular dynamics studies of the RISC^21^.

The post-RISC magnesium-γ is similarly coordinated by nt OP atoms (Fig. 2E) as in previous studies^22^. Computational analyses describe that such nt bound magnesium ions has an enthalpy of −32 kcal/mol^22^. This value is within the average enthalpy of magnesium-γ during the simulations (−30.4 ± 4.1 kca/mol) (Fig. 2G). The pre-RISC magnesium-β exhibits higher enthalpy values (−9.1 ± 3.8 kcal/mol) relative to the lower, enthalpically driven post-RISC magnesium-β (−51.6 ± 3 kcal/mol) (Fig. 2G). Magnesium-β in pre-RISC structures is therefore in a high energy intermediate state during the mRNA hydrolytic cycle. Compared to the other RISC cofactors, magnesium-α maintains the lowest average enthalpy value during the simulations (−97.8 ± 7.7 kcal/mol) (Fig. 2G).

### Magnesium-β of effective antiviral siRNAs are exothermic

**Figure 3:**
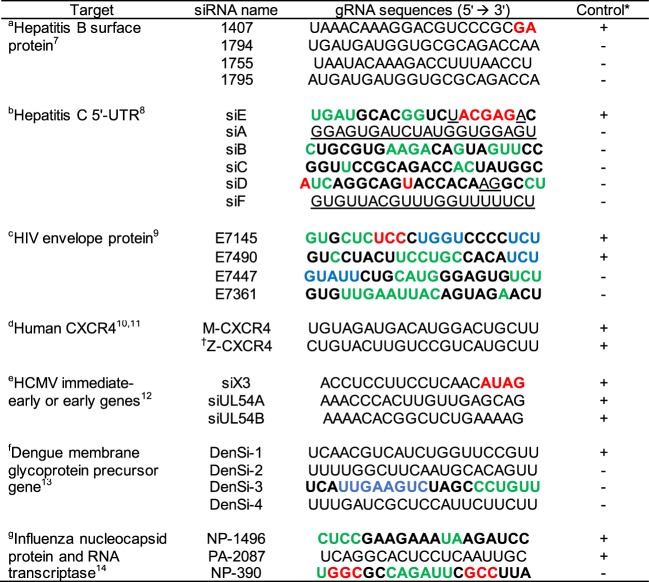
Antiviral positive and negative control gRNA sequences. All published references are numbered under the first column ‘Target’. Reference gene accession numbers are: ^a^AB076678, ^b^AY045702, ^c^U26942, ^d^NM003467, ^e^KX544841, ^f^EU848545 and ^g^MH898433/MH898427. The gRNA structures (excluding terminal overhangs) and target mRNA were designed for *in silico* analyses based on the referenced siRNA studies^7–14^. The color codes describe the target mRNA secondary structure: red = pseudoknot regions; green = hairpin loops; blue = bulges; black = stems; underlined = linker sequence. Note that only HCV^27,28,32^, HIV^33^, Dengue^34^ and influenza nucleocapsid protein^49^ have experimentally verifiable RNA secondary structures. ^†^The gRNA 5′-end target is 5 nt upstream of a mRNA pseudoknot. *The effectivity is noted as: + = positive control gRNAs; − = negative control gRNAs.

The coordination, presence and enthalpy values of RISC cofactors offer unique opportunities to investigate the efficacies of several gRNAs derived from antiviral siRNAs (Fig. 3). These gRNAs, and their corresponding complementary mRNAs, were modeled into the post-RISC structure for stochastic molecular simulations (see *Methods*). The gRNAs (Fig. 3) derived from effective *in vivo* siRNA experiments are termed positive control gRNAs and the less effective, or completely ineffective, siRNAs are termed negative control gRNAs. Statistical analyses on the enthalpy of RISC cofactors between positive and negative control gRNAs show small and negligible effects for magnesium-α (*η*^2^ = 0.019) and magnesium-γ (*η*^2^ = 0.003) (Supplementary Table S4). In contrast, and in support of hypothesis (i), the largest effect between positive and negative control gRNAs occurs in the enthalpy of magnesium-β (*η*^2^ = 0.137; Supplementary Table S4).

**Figure 4:**
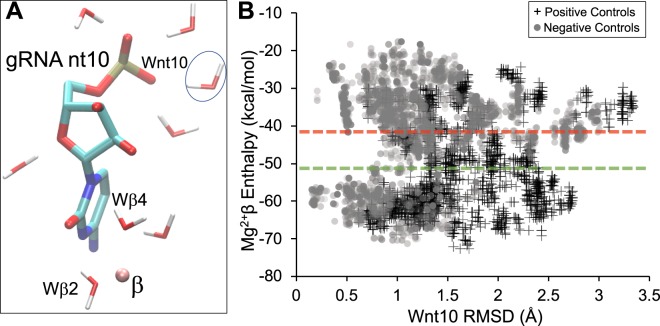
Magnesium-β enthalpy values of gRNAs derived from antiviral siRNAs. Electrostatic hydration of gRNA nt10 (**A**). Magnesium-β and the two inner hydration molecules are noted. The encircled water molecule (Wnt10) deviates most between positive and negative control gRNAs. The molecular simulation results (**B**) for positive (+) and negative (circles) antiviral gRNAs depicts the magnesium-β enthalpy values (y-axis) and the Wnt10 RMSD (x-axis). The dashed lines are the average enthalpy values for the positive (green) and negative (red) control gRNAs.

Subsequent analyses between positive and negative control gRNAs show that small deviations occur for magnesium-β contact molecules (*η*^2^ < 0.02), except for the slightly higher deviation observed by gRNA nt10 (*η*^2^ = 0.05) (Supplementary Table S5). The gRNA nt10 forms electrostatic interactions with several water molecules (Fig. 4A). Only one of these gRNA nt10 water molecules (Wnt10; encircled in Fig. 4A) displays large RMSD differences between positive and negative controls (Fig. 4B) (*η*^2^ = 0.13; Supplementary Table S6). The average magnesium-β enthalpy values between positive (−50.5 ± 11.9 kca/mol) and negative gRNA controls (−40.9 ± 12.1 kca/mol) are indicated (Fig. 4B). Other factors that determine siRNA efficacy were considered since a few false positive (siA, siC, DenSi-2 and DenSi-3) and false negative (Z-CXCR4, siUL54A and Den1) enthalpy values were noted (Fig. 4B).

### Critical seed region accessibility is integral for siRNA efficacy

The siRNA design guidelines by Reynolds *et al*.^6^ solely focus on RNA primary sequences, but are mRNA secondary structures also a factor? If so, what type of mRNA secondary structure, or combinations of structures, might be involved? Pseudoknots were first discovered by Pleij *et al*.^23^ within the RNA 3′-terminus of a few viruses. A typical RNA pseudoknot conformation is an H type (H = hairpin). These H type pseudoknots are comprised of two stem (S) regions connected within three loops (L), labeled as S1-S2 and L1-L3, respectively^24^. To test hypothesis (ii), the full mRNA sequences targeted by the antiviral gRNAs (Fig. 3) were processed for pseudoknot predictions (see *Methods*)^25^.

From the referenced gRNAs, 42% of positive control gRNAs are close to or partly target mRNA pseudoknots (Fig. 3). For instance, the anti-HIV positive control gRNA, E7145, has the final two gRNA nts of the seed region (nt7-nt8) complement a mRNA pseudoknot. The gRNA nt7-nt8 are less critical for initial pre-RISC nt hybridization, thereby facilitating E7145 targeting and the mRNA to unfold. Full gRNA-mRNA hybridization is then formed within Ago2 and mRNA nuclease degradation commences. In a similar way, the anti-hepatitis positive control gRNAs, 1407 and siE, and the anti-HCMV, siX3, complement pseudoknot sequences albeit outside the gRNA seed region. Both anti-CXCR4 positive control gRNAs do not directly target pseudoknots, although a pseudoknot is located 5 nts upstream the mRNA that is targeted by Z-CXCR4 gRNA^11^. In a variation to this theme, the first four nt of the anti-influenza positive control gRNA, NP-1496, target a hairpin loop. These mRNA loop structures not involved in complementary base pairing are also ideal for interactions with the pre-RISC critical seed region (gRNA nt2-nt4; Fig. 3).

The negative control gRNAs siD for anti-HCV and NP-390 for anti-influenza target internal repeats of mRNA pseudoknots (Fig. 3). Avoiding internal repeats is one of the siRNA design guidelines^6^. The NP-390 gRNA^14^ specifically has its critical seed region (nt2-nt4) directly complementing a pseudoknot. These three pre-RISC gRNA nts are essential for initially recognizing the target mRNA^15^. The NP-390 gRNA is therefore rendered ineffective because of the complexity and rigidity in a pseudoknot fold occludes interactions with the critical seed region. The anti-HCV gRNA, siD, is similarly compromised and is discussed in the next section.

### Temporary RISC cofactors are imported from mRNA secondary structures

Although the human activated-RISC structure is not resolved, there is an argonaute crystal structure from *Thermus thermophilus* (TtAgo)^26^ that represents an intermediate stage between the pre- and post-RISC (Fig. 1)^16^. The TtAgo is hybridized to a complementary 19-nt mRNA target sequence with a 21-nt “guide DNA” (gRNA surrogate)^26^. As in the post-RISC, TtAgo possesses three magnesium cofactors at distinct coordinates^26^. Therefore, how is it that magnesium-α and magnesium-γ cofactors are present in activated- and post-RISC structures, but not in pre-RISC structures? Where do these temporary cofactors come from during the RISC catalytic cycle? Egli *et al*.^18^ showed that fifteen magnesium ions stabilize the beet western yellow virus pseudoknot and 42% of the positive control gRNAs are close to or partly incorporate magnesium-rich mRNA secondary structures (Fig. 3). As hypothesis (iii) states, temporary RISC magnesium cofactors are therefore imported during gRNA-mRNA hybridization. Importing these temporary cofactors then primes activated-RISC structures for subsequent mRNA hydrolysis in the post-RISC stage.

**Figure 5:**
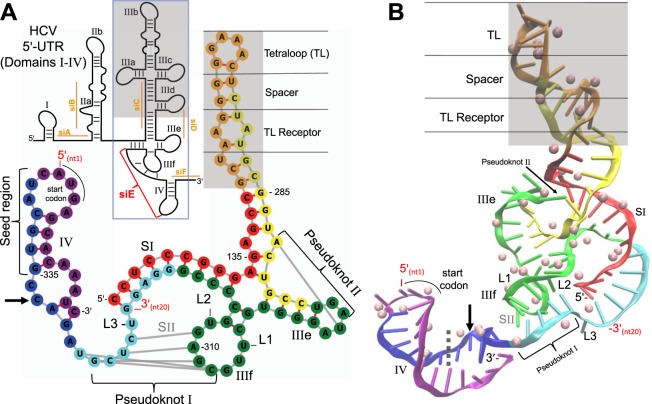
The antiviral RISC targeting the HCV 5′-UTR. The schematic in panel A is the full HCV 5′-UTR with its domains labeled, IRES boxed in and the anti-HCV gRNA (siE) binding region indicated (red). Ineffective anti-HCV 5′-UTR gRNAs, from siRNAs designed by Watanabe *et al*.^8^, are underlined and labeled in orange. The grey enclosed area represents the reduced tetraloop of domain IV. The secondary (**A**) and tertiary (**B**) RNA structures of the HCV 5′-UTR IRES (colored red (5′-end) to purple (3′-end)) show the domains (roman numerals), stems (S) and loops (L) labeled with the complement nt of the pseudoknots (I and II) connected by grey lines (**A**) or bracketed (**B**). The 5′- and 3′-ends of the anti-HCV gRNA (siE) are color-labeled red with the seed region and start codon indicated. The arrow indicates the cleavage site. The pink spheres in panel B are the magnesium ion coordinates (44 in total).

The HCV 5′-UTR mRNA secondary structure, with its pseudoknots, has been known for some time^27^ - shown here as a schematic (Fig. 5A). The HCV 5′-UTR IRES secondary (Fig. 5A) and tertiary structures (Fig. 5B), previously described by Berry *et al*.^28^, depict the main pseudoknot (I), an additional pseudoknot (II), plus other hairpins, stems and loops. The HCV 5′-UTR IRES potentially binds 44 magnesium ions that are concentrated at specific mRNA secondary structures (Fig. 5B). Excluding the tetraloop-bound magnesium ions (16 in total), there are 4 magnesium ions bound within domain IIIe, 6 with domain IIIf, 9 within domain IV, and 6 within SI of pseudoknot (I). The remaining three magnesium ions are pseudoknot-bound. There are 2 magnesium ions in proximity to L3 at the phosphodiester bond of pseudoknot (I) and 1 magnesium ion is intercalated between the complementary nt of pseudoknot (II).

Mapping the anti-HCV gRNAs from Watanabe *et al*.^8^ onto the 5′-UTR IRES structure reveal that negative control gRNAs target stems/hairpins (siB, siC and siD gRNAs) or disordered regions (siA and siF gRNA) (for specifics see highlighted residues in Figs 3 and 5A). Both siA and siF gRNAs map onto disordered, non-complemented mRNA sequences that are ideal for gRNA-mRNA binding, but such mRNA structures lack magnesium ions. The siC and siD gRNAs map onto a highly ordered hairpin and pseudoknot, respectively. Although these are magnesium-rich mRNA structures, access to the critical seed region (gRNA nt2-nt4) is restricted by mRNA complement base pairing (i.e., stems). This is particularly the case for the siD gRNA targeting the internal repeat of pseudoknot (II). The gRNA siD 5′-terminus and its nt10 (the hydrolytic cleavage site) target the two complementary base pairs of pseudoknot (II). As in the anti-influenza, NP-390, such a rigid mRNA secondary structure will occlude initial pre-RISC interactions.

The anti-HCV positive control siE complements mRNA nt323-nt342 with its critical gRNA seed region mapping onto the loop of hairpin IV just before the first base of the HCV 5′-UTR start codon (A). The gRNA seed region is then able to hybridize smoothly with the apical loop of hairpin IV. This positioning consolidates gRNA-mRNA interactions such that the 5′-phosphodiester bond of mRNA nt333 becomes the primary hydrolytic cleavage site. Thereafter, nt14-nt18 of the gRNA maps directly onto pseudoknot (I) that complements interior loop IIIf. More importantly, the targeted HCV mRNA sequence incorporates 10 magnesium ions, with 4 ions within the seed region itself (Fig. 5B). Therefore, the target mRNA is optimal for initial critical seed region interactions and enables the RISC catalytic cycle by importing its temporary cofactors.

## Discussion

**Figure 6:**
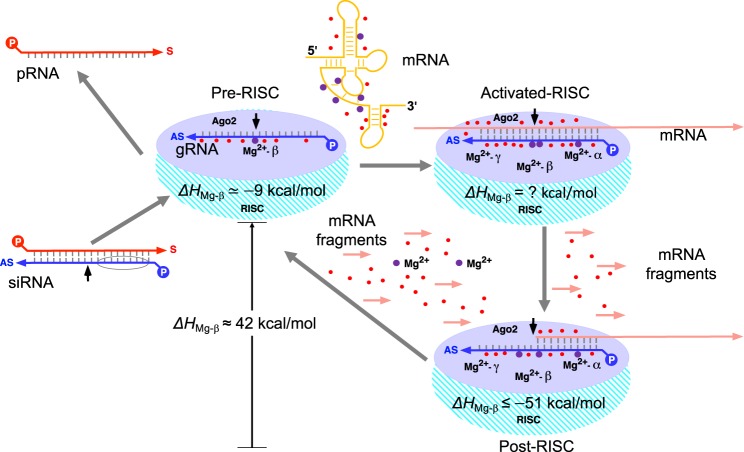
A simple schematic on the stages of the RISC catalytic cycle. Ejection of the pRNA (red strand) from the siRNA establishes the pre-RISC with its associated gRNA (blue strand), water molecules (red circles) and magnesium-β (dark purple circles). The siRNA seed region is encircled and the mRNA cleavage site is indicated by the arrow. Targeting magnesium-rich (dark purple circles) mRNA structures (light orange) enables the activated-RISC by importing temporary cofactors for mRNA hydrolysis. The enthalpy (*ΔH*) of the activated-RISC cofactors are currently undetermined. Increasing enthalpy after releasing post-RISC mRNA fragments (short light orange arrows) and cofactors re-establishes the start of the catalytic cycle (pre-RISC).

An optimal gRNA-mRNA hybridization is initiated when the pre-RISC critical seed region (nt2-nt4) targets mRNA structures not involved in intramolecular base pairing. Magnesium-rich mRNA pseudoknots and/or hairpins are ideal targets downstream of the gRNA critical seed region (Fig. 3). These highly-ordered mRNA structures are theoretically responsible for importing magnesium-α and magnesium-γ (Fig. 6). At the activated-RISC stage, RNA base pairing is enthalpically driven and entropically favored, but its free energy is disfavored^29^. During the activated-RISC stage, magnesium-β and magnesium-γ are in close proximity^26^ and responsible for cleaving the target mRNA via an electrostatic catalytic effect^30^ After mRNA cleavage, the thermodynamics shift during the post-RISC stage to a favorable free energy due to phosphodiester bond hydrolysis^31^. The concomitant dissociation of mRNA fragments and the escape of the temporary cofactors upon returning to the pre-RISC stage causes an extreme exothermic loss in magnesium-β (Fig. 6), The RISC catalytic cycle is completed by compensating through a gain in system entropy.

The process of RNAi is multifaceted that requires extra level siRNA design guidelines. For instance, the original guideline by Reynolds *et al*.^6^ to avoid internal repeats is too general. The ineffectiveness of including internal repeats is clearly indicated by the anti-HCV (siD) and anti-influenza (NP-390) siRNAs that target the complemented nt forming a pseudoknot (Fig. 3). An extra level guideline will therefore specifically indicate that targeting loops of magnesium-rich hairpins and/or pseudoknots is favorable to activate the RISC catalytic cycle. The hairpin loop targeted by the gRNAs of anti-influenza NP-1496 and anti-HCV siE are prime examples for initial pre-RISC critical seed region interactions (Fig. 3). The siE gRNA also targets a magnesium-rich pseudoknot downstream of its seed region (Fig. 3). Another specific siRNA design guideline is shown by the exothermicity of magnesium-β.

The stochastic molecular simulations show that magnesium-β interactions are generally more favorable for effective antiviral gRNAs than for their negative control counterparts (Fig. 4B). The presence of false positives indicates that mRNA hydrolysis will not take place for structural reasons even if post-RISC magnesium-β interactions are favorable. On the one hand, the false positives anti-HCV, siC, and the anti-Dengue, DenSi-3, target mRNA complementary base paired stem regions that will block interactions with the gRNA critical seed region (Fig. 3). On the other hand, targeting less complex or disordered mRNA structures also deters mRNA hydrolysis, as is with the false positive anti-HCV gRNA, siA (Fig. 3). These disordered mRNA structures lack chelated magnesium ions to import for an activated-RISC. The situation is logically *vis versa* for the false negative gRNA Z-CXCR4.

Both anti-CXCR4s are effective siRNAs^10,11^, but the post-RISC magnesium-β of M-CXCR4 is more favorable (−52 ± 6 kcal/mol) than the false negative, Z-CXCR4 (−43 ± 3 kcal/mol). The mRNA target of Z-CXCR4, however, actually possesses a pseudoknot five nt upstream (Fig. 3). Regarding the anti-HCMV false negative, siUL54A, the post-RISC magnesium-β enthalpy (−34 ± 3 kcal/mol) reflects its siRNA activity since siUL54A is experimentally less effective^12^ than the enthalpically driven siX3 (−57 ± 5 kcal/mol). Although *in silico* methods indicate siX3 binds a mRNA pseudoknot at its 3′-terminus (Fig. 3), the HCMV RNA structure is not known to determine the siUL54A target. Only a few RNA secondary structures used in this study have been experimentally resolved^27,28,32–34^. Designing more effective siRNA will therefore benefit by combining the thermodynamics of post-RISC magnesium-β interactions and advanced methods that resolve RNA structures^35^.

All the foregoing data and insights into the RISC catalytic cycle (Fig. 6) dictate that future siRNA designs should ensure that gRNAs selectively target mRNAs at specific secondary structures and benefit from enthalpically favorable interactions with post-RISC magnesium-β. The resulting extra level siRNA design guidelines are specifically summarized by:

1. Assuring access of gRNA critical seed region (nt2-nt4) for initial mRNA binding,
2. Targeting magnesium-rich pseudoknot and/or hairpin loops, and
3. Maintaining a post-RISC magnesium-β enthalpy range of −72 to −51 kcal/mol.

## Methods

### Preparation of the RISC system

Both RISC crystal structures by Schirle *et al*.^16^ (PDB: 4W5N and 4W5O) were prepared and optimized for this study. The pre-RISC structure (PDB: 4W5N) includes the gRNA strand (nt1-nt7 and nt12-nt20), 1 magnesium cofactor (β) and 34 water molecules. The post-RISC structure (PDB: 4W5O) includes a gRNA strand (nt1-nt17 and nt21), a complementary 3′ portion of the target mRNA (nt1-nt9), 3 magnesium cofactors (α, β and γ), and 433 water molecules. There are fewer water molecules in pre-RISC than in post-RISC. The 433 post-RISC water molecules were therefore substituted into the pre-RISC assuring proper hydration of magnesium-β. The chain identities for Ago2 (A), magnesium cofactors (B), waters (C), gRNA (D) and mRNA (E) were changed as indicated.

The Ago2 has missing loops (residues 89–90, 121–126, 267–275, 297–305 and 822–835) that were modeled using Chimera’s Modeller^36^. The Genesilco’s ModeRNA server^37^ was used to model the full-length gRNA (21 nts) using a composite template from the post-RISC structure by Schirle *et al*.^16^. These modeled gRNA and mRNA were further used as templates to model the respective RNA strands of the antivirals in Fig. 3. The ModeRNA server^37^ was also used to compose the HCV 5′-UTR using the crystal structure PDB: 3TB4 due to its low resolution. The missing RNA hairpin of domain IV was constructed from PDB:1J2B and the positioning of the magnesium cofactors were implemented by the Monte Carlo Tightly Bound Ion Model server^38^.

X-Ray crystal structures have unresolved hydrogen atoms, so each chain of the RISC structures was prepared and optimized separately using the Schrödinger’s Maestro Protein Preparation Wizard^39^. Optimization at pH 7 resulted in 21 protonation states of His (20) and Lys (1) residues for the post-RISC (PDB: 4W5O) and 23 protonation states of His (20) and Lys (3) residues for the pre-RISC (PDB: 4W5O). Each prepared and optimized chain was minimized separately and then merged as a ternary structure. Steric clashes were detected and eliminated via local minimization.

Each RISC system was then subjected to several global minimizations and the last three minimizations were used to generate structurally distinct replicates (Rep1, Rep2 and Rep3). All minimizations in this preparation stage were performed using the Maestro default setting. Regardless of the aforementioned preparations, optimizations and minimizations, the replicate structures relatively maintained their respective coordinates from the original PDB files. All tertiary structural images were captured using VMD^40^.

### Stochastic molecular simulations

The molecular simulations were generated using the Metropolis Monte Carlo-based Protein Energy Landscape Exploration server (PELE)^41^. The PELE server can be accessed at pele.bsc.es and its many applications have been previously explained^41,42^. Briefly, the PELE software performs three steps in its algorithm: (1) protein and ligand local perturbation, (2) amino acid side chain sampling, and (3) a global minimization of the entire system. These steps are repeated for a desired number of iterations and are completed within 24 h. For this study, a protein motion ready-made script was used for 400 iterations with a single cpu. Alterations were made to the PELE protein motion ready-made script are in Supplementary Text S1. The Metropolis Monte Carlo-based method employed by PELE accepts an iteration if the enthalpy of evaporation (*ΔH*) is equal to or less than the initial value. An iteration is rejected if the enthalpy determined is greater than the initial value. Enthalpy values (kcal/mol) of the RISC system were recorded during the simulations.

To calculate enthalpy, the PELE software uses the force field known as the optimized potentials for liquid simulations (OPLS-2005)^43^ that was shown suitable for metals^44^. The OPLS-2005 treats metals as ions interacting via ionic bonding. Magnesium cofactors were constrained for Rep1-Rep3 at their initial position throughout the simulations at 1.5 kcal/mol. For the magnesium cofactors, PELE calculates the magnesium binding enthalpy according to Eq. (1) as follows:

1 $$\Delta H=\Delta {H}_{ab}-(\Delta {H}_{a}+\Delta {H}_{b})$$

where ${H}_{{ab}}$ is the enthalpy for the entire RISC system, including the magnesium cofactors, and ${H}_{a}$ and ${H}_{b}$ are their separate enthalpy values. The simulations resulted in approximately 300 frames (i.e., accepted iterations) per replicate for all simulations. The first 100 frames were deleted since the system reaches a relatively steady enthalpy state at this stage during the simulations.

### Pseudoknot predictions

Several algorithms^25,45–47^ were tested for pseudoknot predictions using, as controls, the 5′-UTR pseudoknot of HCV, several other resolved pseudoknot structures (PDBs: 1L2X, 5D5L and 4PQV), and the hairpin (PDB: 1J2B) as a negative control. The Dotknot server^25^ was the most consistent in representing controls based on two Dotknot criteria that represent the pseudoknot position(s): (1) the best free energy to nt length ratio, and (2) the global structure. The full mRNA target sequences of the antiviral siRNAs in Fig. 3 were processed using the DotKnot server^25^ and determined based on these two criteria. The secondary structure of the HCV 5′-UTR was visualized and saved using the ViennaRNA web-application, *forna*^48^. Note that DotKnot did not accurately represent the global RNA secondary structures (i.e., positions of linkers, hairpins, bulges and stems) - only the specific nts that form the pseudoknot. The positions of RNA secondary structures represented in Fig. 3 were referenced studies for HCV 5′-UTR^27,28,32^, HIV envelope protein^33^ and influenza nucleocapsid protein^49^. To the knowledge of the authors, these resolved RNA secondary structures are the only ones available for the sequences used in this study (Fig. 3).

### Statistics

The effect sizes from the data were calculated using Cohen’s^50^ eta squared (*η*^2^) according to Eq. (2) as follows:

2 $${\eta }^{2}=\frac{Treatment\,Sum\,of\,Squares}{Total\,Sum\,of\,Squares}$$

Cohen’s *η*^2^ determines if the difference between the independent variables (i.e., positive versus negative controls) is small (0.01), medium (0.059) or large (0.138), regardless of the *p-value*. The central limit theorem validates normal-based methods for large samples sizes^51^, i.e., the PELE simulations (~200 frames by 3 Reps). Since negligible differences occured between replicates (Supplementary Table S1) the averages of the combined Reps from the PELE simulations were used to calculate the ANOVA (Supplementary Tables S2–S4). Similar methods were previously used to statistically analyze stochastic molecular simulations^52,53^.

## Supplementary information

Supplementary Material
