# Supplementary material for: New opportunities for designing effective small interfering RNAs
Source: Sci Rep. 2019 Nov 6;9:16146. doi: 10.1038/s41598-019-52303-5 (PMC6834666; doi:10.1038/s41598-019-52303-5)
Supplement: Supplementary file 1 — Supplementary Material [file 41598_2019_52303_MOESM1_ESM.pdf]

# New opportunities for designing effective small interfering RNAs

James J. Valdés[*,1,2] and Andrew D. Miller[*,2,3]

[1]*Institute of Parasitology, Biology Centre, Czech Academy of Sciences, Branišovska 1160/31, CZ-37005, České Budějovice, Czech Republic*

[2]*Veterinary Research Institute, Hudcova 70, CZ-62100, Brno, Czech Republic*

[3]*KP Therapeutics Ltd, 86 Deansgate, Manchester, M3 2ER, UK*

*Corresponding authors: J.J.V. (email: valdjj@gmail.com), A.D.M. (email: miller@vri.cz)

Supplementary Figure

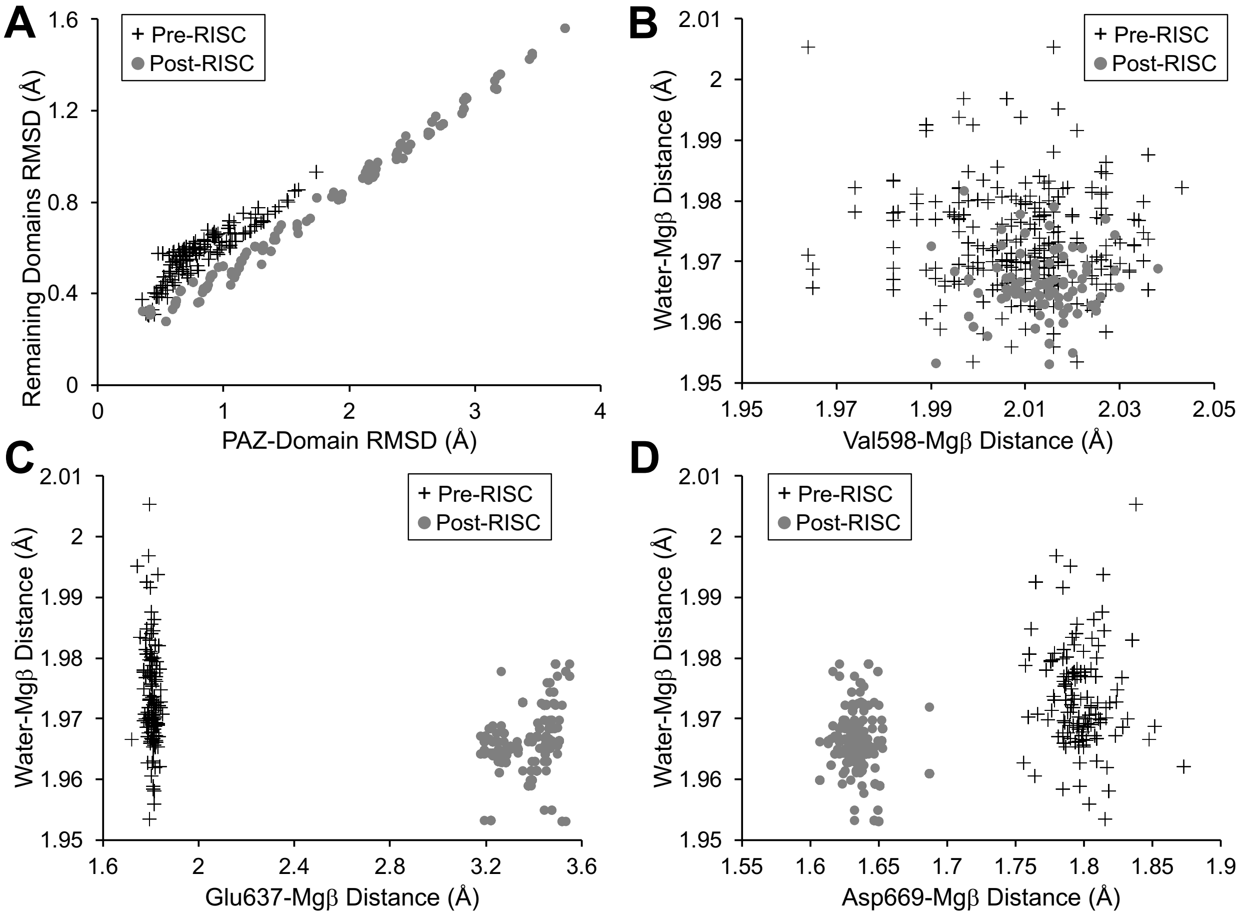


Supplementary Figure S1. Pre- and post-RISC dynamics. (A) The RMSD of the PAZ-domain (x-axis) and of the remaining Ago2 domains (y-axis). (B-D) The average inner shell water distances (y-axis) and specified Ago2 residues (x-axis) with magnesium-β.

Supplementary Tables S1-S6 for descriptive statistics - ANOVA

Supplementary Table S1. The total enthalpy for each Reps of the pre- and post-RISCs

| Treatment | Sum of Squares | df | Variance | F | P |
|---|---|---|---|---|---|
| Pre-RISC | | | | | |
| Between Reps1-3 | 17536.4 | 2 | 8768.2 | 2.8 | 0.06 |
| Within Reps1-3 | 2775803.8 | 884 | 3140.1 | | |
| Total | 2793340.2 | 886 | 3153 | | |
| Post-RISC | | | | | |
| Between Reps1-3 | 77023.8 | 2 | 38511.9 | 17.1 | 0.00 |
| Within Reps1-3 | 1905564.0 | 848 | 2247.1 | | |
| Total | 1982587.8 | 850 | 2332 | | |

Supplementary Table S2. The RMSD and total enthalpy of the pre- and post-RISC

| Treatment | Sum of Squares | df | Variance | F | P |
|---|---|---|---|---|---|
| RMSD | | | | | |
| Between RISCs | 432.3 | 1 | 432.3 | 1074.4 | 0.00 |
| Within RISCs | 694.9 | 1727 | 0.40 | | |
| Total | 1127.2 | 1728 | 0.65 | | |
| Enthalpy | | | | | |
| Between RISCs | 3664347154.3 | 5 | 732869430.9 | 271145.1 | 0.00 |
| Within RISCs | 4681367.8 | 1732 | 2702.9 | | |
| Total | 3669028522.1 | 1737 | 2112279.0 | | |

Supplementary Table S3. RMSD of magnesium-β shell molecules (pre- and post-RISC)

| Treatment | Sum of Squares | df | Variance | F | P |
|---|---|---|---|---|---|
| Mgβ 1$^{st}$ shell | | | | | |
| Between controls | 0.93 | 1 | 0.93 | 69.5 | 0.00 |
| Within controls | 17.2 | 1286 | 0.013 | | |
| Total | 18.1 | 1287 | 0.014 | | |
| Mgβ 2$^{nd}$ shell | | | | | |
| Between controls | 0.34 | 1 | 0.34 | 22.9 | 0.00 |
| Within controls | 19.1 | 1286 | 0.02 | | |
| Total | 19.4 | 1287 | 0.015 | | |

Supplementary Table S4. The magnesium enthalpy for positive and negative gRNA controls

| Treatment | Sum of Squares | df | Variance | F | P |
|---|---|---|---|---|---|
| Magnesium-α | | | | | |
| Between controls | 316997.1 | 1 | 316997.1 | 346.2 | 0.00 |
| Within controls | 16047425.7 | 17526 | 915.6 | | |
| Total | 16364422.8 | 17527 | 933.7 | | |
| Magnesium-β | | | | | |
| Between controls | 397987.3 | 1 | 397987.3 | 2769.2 | 0.00 |
| Within controls | 2518825.6 | 17526 | 143.7 | | |
| Total | 291681.9 | 17527 | 166.4 | | |
| Magnesium-γ | | | | | |
| Between controls | 7303.4 | 1 | 7303.4 | 47.5 | 0.00 |
| Within controls | 2693847.1 | 17526 | 153.7 | | |
| Total | 2701150.5 | 17527 | 154.1 | | |

Supplementary Table S5. RMSD of magnesium-β shell molecules for positive and negative gRNA controls

| Treatment | Sum of Squares | df | Variance | F | P |
|---|---|---|---|---|---|
| Inner shell | | | | | |
| Between controls | 0.214 | 1 | 0.214 | 2.27 | 0.13 |
| Within controls | 1648.3 | 17526 | 0.094 | | |
| Total | 1648.5 | 17527 | 0.094 | | |
| Outer shell | | | | | |
| Between controls | 44.9 | 1 | 44.9 | 301.1 | 0.00 |
| Within controls | 2614.1 | 17526 | 0.149 | | |
| Total | 2659.0 | 17527 | 0.152 | | |
| Asp597 | | | | | |
| Between controls | 4.3 | 1 | 4.3 | 94.0 | 0.00 |
| Within controls | 791.8 | 17526 | 0.046 | | |
| Total | 796.1 | 17527 | 0.045 | | |
| Val598 | | | | | |
| Between controls | 6.75 | 1 | 6.75 | 158.1 | 0.00 |
| Within controls | 747.84 | 17526 | 0.0427 | | |
| Total | 754.59 | 17527 | 0.0431 | | |
| Glu637 | | | | | |
| Between controls | 9.65 | 1 | 9.65 | 321.03 | 0.00 |
| Within controls | 526.99 | 17526 | 0.030 | | |
| Total | 536.64 | 17527 | 0.031 | | |
| Asp669 | | | | | |
| Between controls | 1.14 | 1 | 1.14 | 55.66 | 0.00 |
| Within controls | 360.34 | 17526 | 0.021 | | |
| Total | 361.48 | 17527 | 0.021 | | |
| gRNA nt10 (C1') | | | | | |
| Between controls | 78.2 | 1 | 78.2 | 934.23 | 0.00 |
| Within controls | 1466.5 | 17526 | 0.084 | | |
| Total | 1544.7 | 17527 | 0.088 | | |
| gRNA nt11 (C1') | | | | | |
| Between controls | 21.75 | 1 | 21.75 | 110.41 | 0.00 |
| Within controls | 3451.89 | 17526 | 0.197 | | |
| Total | 3473.64 | 17527 | 0.198 | | |

Supplementary Table S6. RMSD of gRNA nt10 water molecule (pre- and post-RISC)

| Treatment | Sum of Squares | df | Variance | F | P |
|---|---|---|---|---|---|
| Wnt10 | | | | | |
| Between controls | 977.32 | 1 | 977.3 | 2601.0 | 0.00 |
| Within controls | 6585.38 | 17526 | 0.3758 | | |
| Total | 7562.70 | 17527 | 0.4315 | | |

Supplementary Text S1 on the alterations to the PELE ready-made script for protein motion:

```
constraints to_current _MG_ B:900 B:902 0.5 #The chain:id range to maintain the current position of the ligand 3 magnesium ions

pele &
	het B:900 & #The chain:id of the ligand 3 separate for magnesium ions
	het B:901 &
	het B:902 &
	pdbmodel yes &
	init_min no & #Do not produce an initial minimization before the simulation run
	task &
		show bind_ene 1 & #Records the enthalpy of the 3 magnesium ions
		show bind_ene 2 &
		show bind_ene 3 &
		exit steps gt 400 & #Increased for more sampling
	end_task &
	temp 2000 &
	anmfreq 3 &
	spfreq 3 &
	mifreq 3 &
	wrfreq 1 &
	side &
		randomize no &
		verbose no &
		failsafe no &
		sideend &
	path traj_ &
	min &
		rmsg 0.04 &
		nbup yes &
		gbup yes &
		alphaup yes &
	minimend &
	caconst 0.25 & #The remaining alterations produced favorable ANM perturbations based on experimental conformations
	rem_bulk_mov 3 &
	anm_eig_freq 100000 &
	anm_altm_freq 10 &
	anm_altm_type 4 &
	lanmanm neig 6 &
	lanmanm move_ca 0.5 &
	lanmanm mix_modes 0.80 &
	lanmanm omit_no A:805 A:811 & #Were omitted from ANM due to large conformational changes
	lanmmin &
		mxitn 100 &
		iter 1 &
		rmsg 0.04 &
		nbup yes &
		alphaup no &
	minimend
```
